# The impact of community-based non-pharmacological interventions on cardiovascular and kidney disease outcomes in remote dwelling Indigenous communities: A scoping review protocol

**DOI:** 10.1371/journal.pone.0269839

**Published:** 2022-06-10

**Authors:** Ikechi G. Okpechi, Vinash Kumar Hariramani, Naima Sultana, Anukul Ghimire, Deenaz Zaidi, Shezel Muneer, Mohammed M. Tinwala, Feng Ye, Megan Sebastianski, Abdullah Abdulrahman, Branko Braam, Kailash Jindal, Maryam Khan, Scott Klarenbach, Soroush Shojai, Stephanie Thompson, Aminu K. Bello

**Affiliations:** 1 Division of Nephrology and Immunology, Faculty of Medicine and Dentistry, University of Alberta, Edmonton, Alberta, Canada; 2 Division of Nephrology and Hypertension, University of Cape Town, Cape Town, South Africa; 3 Kidney and Hypertension Research Unit, University of Cape Town, Cape Town, South Africa; 4 Knowledge Translation Platform, Alberta SPOR SUPPORT Unit Department of Pediatrics, University of Alberta, Edmonton, Canada; Università degli Studi di Milano, ITALY

## Abstract

**Introduction:**

Indigenous people represent approximately 5% of the world’s population. However, they often have a disproportionately higher burden of cardiovascular disease (CVD) risk and chronic kidney disease (CKD) than their equivalent general population. Several non-pharmacological interventions (e.g., educational) have been used to reduce CVD and kidney disease risk factors in Indigenous groups. The aim of this paper is to describe the protocol for a scoping review that will assess the impact of non-pharmacological interventions carried out in Indigenous and remote dwelling populations to reduce CVD risk factors and CKD.

**Materials and methods:**

This scoping review will be guided by the methodological framework for conducting scoping studies developed by Arksey and O’Malley. Both empirical (Medline, Embase, Cochrane Library, CINAHL, ISI Web of Science and PsycINFO) and grey literature references will be assessed if they focused on interventions targeted at reducing CVD or CKD among Indigenous groups. Two reviewers will independently screen references in consecutive stages of title/abstract screening and then full-text screening. Impact of interventions used will be assessed using the reach, effectiveness, adoption, implementation, maintenance (RE-AIM) framework. A descriptive overview, tabular summaries, and content analysis will be carried out on the extracted data.

**Ethics and dissemination:**

This review will collect and analyse evidence on the impact of interventions of research carried out to reduce CVD and CKD among Indigenous populations. Such evidence will be disseminated using traditional approaches that includes open-access peer-reviewed publication, scientific presentations, and a report. Also, we will disseminate our findings to the government and Indigenous leaders. Ethical approval will not be required for this scoping review as the data used will be extracted from already published studies with publicly accessible data.

## Introduction

Cardiovascular diseases (CVDs) are the leading cause of global mortality and a major contributor to reduced quality of life. The Global Burden of Disease study of 2019 showed that CVD risk factors were the leading causes of death: systolic blood pressure accounted for 10.8 million (95% uncertainty interval [UI]: 9.5–12.1) deaths (19.2% [16.9–21.3]) followed by tobacco which accounted for 8.7 million (95% UI: 8.1–9.3) deaths (15.4% [14.6–16.2]) [1]. The burden of several CVD risk factors, including diabetes mellitus, high blood pressure (BP), and over-weight and obesity have been increasing worldwide. For instance, in 2010, the global prevalence of hypertension increased to 31.1% (95% CI: 30.0–32.2) in the global adult population (1.38 billion people), from 25.9% (24.4–27.5) in 2000 [2]. In absolute numbers, this represented an increase from 924.2 million to 1.38 billion people over the same period and was noted to be much higher in resource-limited countries (31.5%, 1.04 billion) than in wealthy countries (28.5%, 349 million) [2]. Elevated BP is also associated with increased premature mortality and in 2015, one study showed that global all-cause mortality associated with systolic BP ≥110–115 mmHg was 10.7 million (19.2% of all deaths) [3]. Similarly, the International Diabetes Federation also estimated that mortality associated with diabetes in 2017 to be approximately 5.0 million (95%CI 4.0–6.4) among people aged 20–99 years; thus, accounting for 9.9% of the global all-cause mortality in those within the age range [4]. Increases in other CVD risk factors have also been reported including overweight and obesity [5], physical inactivity [6], and increased consumption of alcohol [7]. Although the prevalence of high cholesterol [8] and smoking [9] have been shown to be declining due to increased population and ageing, they continue to add to the burden of CVDs.

The rising burden of CVD risk factors is also linked with the global increase of chronic kidney disease (CKD). Between 1990 and 2013, the prevalence of CKD increased by 48%, from 318.7 million to 471.9 million (about 2.1% per year) [10]. In the same period, it was noted that the change in number of CKD cases from diabetes and hypertension increased by 82.5% and 26.8%, respectively [10]. Although observational studies show that the prevalence of CKD depends on how it is defined, one systematic review found a high estimated global prevalence of CKD to be between 11 to 13% and an association between CKD and age, diabetes and hypertension [11]. About 1.2 million premature deaths occur each year in kidney disease patients who need dialysis [12], most of the deaths occurring due to lack of access to kidney replacement therapy (KRT). The number of deaths has been estimated to rise to 3.2 million as a result of diabetes and elevated BP [12].

There are more than 5,000 distinct Indigenous groups in 90 countries around the world, representing more than 5% of the world’s population (approximately 370 million people) [13]. However, in countries with Indigenous people, they are often the most disadvantaged, vulnerable and often carry a higher burden of CVD risk and CKD than the general population [14–18]. Despite lack of recent data, one systematic review on the prevalence of non-communicable diseases (NCDs) in Papua New Guinea concluded that there is a strong suggestion that the prevalence of NCDs, particularly type 2 diabetes mellitus and other NCDs such as hypertension, overweight, and obesity, has increased in the largely Indigenous population [19]. Higher CV and kidney disease burden has been associated with socioeconomic disadvantage, erosion of trust, lack of social support and educational opportunities, and difficulties with access to healthcare or affordable prescription medications [20]. A study of Indigenous people in Canada, found CKD (stage 3–5) prevalence to be 7.0% with 80% of CKD patients having at least 1 CVD risk factor (71.6% had diabetes, 70.6% had dyslipidaemia, and 59.1% had hypertension with all 3 present in 38.5%) [18]. In comparison, data from a nationally representative survey in Canada found stage 3–5 CKD prevalence to be 3.1% with 72.3% of adults with CKD having neither hypertension or diabetes [21]. Both CVD and CKD related mortality in Indigenous populations have also been demonstrated to be excessively high [16, 22].

Interventions targeted at early identification or reduction in CVD risk or CKD progression in Indigenous populations have been shown to be well embraced and/or effective [23–26]. Such interventions can be generically grouped as nurse or community-health worker led, outreach programs, mobile screening and treatment units, lifestyle or behavioural changes, telehealth, educational, and interventions focussed on counselling and home visits. The Stop Atherosclerosis in Native Diabetics Study (SANDS) conducted in American Indians showed that aggressive lipid targets could be safely maintained in Indigenous peoples with diabetes with the help of standardized algorithms, point-of-care lipid testing, and non-physician providers [27]. In a New Zealand study that randomized Māori and Pacific patients to a nurse-led community based intervention or standard of care for BP control, there was significantly lower systolic BP and urine protein excretion rate in the nurse-led group than in standard of care patients [28]. Not all such interventions have shown efficacy as one Canadian study that randomized remote and First Nations patients to active treatment (with hypertension specific management short message service [SMS]) or passive treatment (health behaviours SMS alone) to assess the difference in BP reduction between both groups [26]. The active hypertension specific SMS did not lead to improvements in BP control.

A recent scoping review assessed interventions to improve clinical outcomes in Indigenous or remote patients with CKD with a primary objective of identifying evidence-based interventions in such populations [29]. They identified 32 studies that included multidisciplinary (34.4%), satellite (32.3%), telehealth (25.0%), or other (9.4%) interventional types and reported that interventions were more likely to be successful when the remote or Indigenous community was included in program development, with a culturally safe approach [29]. Also, an Australian study that assessed how effective cardiovascular programs were for Indigenous Australians found that the common features of effectiveness of programs were integration of programs within existing services, provision of culturally appropriate delivery models with a central role for Indigenous health workers, and provision of support processes for communities such as transportation [14]. However, the authors noted that the programs were modelled on interventions based on conventional views and did not have strategies that combined traditional knowledge with the delivery of healthcare [14].

The reach, effectiveness, adoption, implementation, and maintenance (RE-AIM) framework provides a practical means of evaluating the impact of health interventions (i.e. programs, policy, and practice) by considering both internal and external validity factors [30, 31]. This framework measures the reach of the target population and efficacy/effectiveness through program outcomes at the individual level, adoption and implementation of the program by personnel and settings at the organizational level, and maintenance of change at both the individual and organizational levels [30]. The RE-AIM framework has been applied in diabetes intervention programs to assess the management of hard-to-reach populations [32], the effectiveness of self-management support programs [33], diabetes learning healthcare programs [34] and in programs aimed at identifying effective practices in diabetes prevention [35]. The framework has also been used to evaluate stroke programs [36], left ventricular assist device programs [37], CVD risk reduction interventions [38] and interventions in dialysis and health system changes in CKD patients [39, 40]. In one study, culturally adapted CVD risk reduction interventions modelled after a specific program were found to be acceptable to Indigenous populations leading to recommendations made for cultural adaptations to include traditional American Indian (AI) exercise activities and AI foods [38]. The aim of this study is to use the RE-AIM framework to assess the impact of non-pharmacological interventions utilized to reduce CVD risk factors and CKD in Indigenous and remote dwelling populations.

## Review question

Can a model of community-based non-pharmacological intervention reduce cardiovascular and/or kidney disease risk factors in Indigenous people living in remote communities?

## Materials and methods

We will be guided by the methodological framework for conducting scoping studies developed by Arksey and O’Malley in 2005 [41]. This framework provided an excellent foundation for scoping study methodology but has been further enhanced by work done by others [42–44]. This framework will include five steps (with an optional sixth step): (1) identifying the research question; (2) identifying the relevant studies; (3) study selection; (4) charting the data and (5) reporting the results; (6) consultation (optional). We will also utilize best practices for conducting and reporting systematic reviews (i.e., Preferred Reporting Items for Systematic Reviews and Meta-Analyses (PRISMA) for Protocols and Scoping Reviews (PRISMA-ScR) for reporting our findings [45, 46].

### Study selection

We will include studies that provide report on interventions with the aim of preventing or reducing the burden of CVD and/or CKD in an Indigenous population. We will group the interventions by the predominant theme of program utilized such as educational, telehealth, healthcare worker (e.g., nurse, pharmacist, community health worker, etc), nutritional, exercise, organizational/facility-based, multi-faceted, and culturally appropriate. We will also categorize studies by the Indigenous population and by country. Two reviewers will independently screen all identified citations for potential inclusion. When agreement on a citation cannot be reached between the two reviewers, a third reviewer will be consulted for resolution. We will conduct this review in 2-stages to screen and select references. The first stage will involve screening of the title/abstract. In this stage, studies will be eligible for full text screening if:

- It is conducted in a recognized Indigenous population. Indigenous populations will be defined as communities that live within, or are attached to, geographically distinct traditional habitats or ancestral territories, and who identify themselves as being part of a distinct cultural group, descended from groups present in the area before modern states were created and current borders defined. They generally maintain cultural and social identities, and social, economic, cultural and political institutions, separate from the mainstream or dominant society or culture [47].
- It uses an intervention designed to improve delivery of care by addressing reduction of CVD and/or CKD risk in the same population
- Publication date (no restriction)
- It is published in English language

In the second stage, full texts, having met the above criteria will then be obtained for further screening. Full texts will be included if:

- It provides a clear definition / description of the type of intervention that was used in the study (e.g., nurse-led, nutritional program, exercise programs, etc).
- It reported a CVD or CKD outcome of interest (Table 1)

**Table 1:** Definition of outcome measures.

Outcome	Description
**A). Cardiovascular disease related outcomes**	
i). Behavioural risk factors	• Reduced smoking
	• Improved dietary choices (e.g., reduced salt intake, increased fruits and vegetables intake, etc)
	• Increased physical activity
	• Reduced alcohol consumption
ii). Clinical risk factors	• Reduced weight / BMI
	• Reduced BP (systolic and /or diastolic)
iii). Biochemical risk factors	• Improved glycaemic index (blood glucose, HbA1c)
	• Reduced serum lipids
**B). Kidney disease-related outcomes**	• Improvement in urine protein / albumin excretion rate
	• Improvement of serum creatinine and/or estimated glomerular filtration rate [eGFR]

Abbreviations: BMI—body mass index; BP—blood pressure; HbA1c—glycated hemoglobin; eGFR—estimated glomerular filtration rate.

The intervention will be defined as “telehealth” if it used any electronic information or telecommunication technologies systems as intervention, “healthcare worker” if the intervention involved the use of a particular type of healthcare worker (e.g. nurse, pharmacist, community health worker, etc), “educational” if the intervention involved some an educational or learning process, “exercise” if the intervention involved a component of increased physical activity, “nutritional” if the intervention involved modification of the subjects diet, “organizational / facility-based” if there are changes made to the structure or organization of the health facility to improve care (e.g. appointment reminders, appointment scheduling, treatment protocol updates prior to intervention, etc), “multi-faceted” if any combinations of these interventions are utilized, and “culturally appropriate” if the intervention methods incorporated Indigenous cultures (e.g. Indigenous methods of meal preparation, embedded Indigenous healthcare worker, etc).

We will assess and report significant and non-significant changes in behavioural (smoking, improved dietary choices, increased physical activity and reduced alcohol consumption), clinical (reduced blood pressure and weight / body mass index), and biochemical (improved glycaemic index and reduced serum lipids) CV risk factors. Similarly, significant and non-significant improvements in biochemical kidney disease outcomes (reduced urine protein excretion and improvements in serum creatinine and/or estimated glomerular filtration rate [eGFR]) will also be reported. To summarize, the inclusion criteria will therefore include:

- **Population**: Remote dwelling Indigenous communities (defined as Indigenous populations who dwell in rural areas.
- **Intervention types**: exercise, nutritional/diet-based, educational, telehealth, healthcare worker, organizational / facility-based, multi-faceted (if combination of interventions is used), and culturally appropriate interventions.
- **Comparator**: comparisons between intervention and control group or baseline results for single group pre-test and post-test studies.
- **Outcomes**: changes in behavioral (smoking, improved dietary choices, increased physical activity, and reduced alcohol consumption), clinical (reduced blood pressure and weight / body mass index), and biochemical (improved glycaemic index and reduced serum lipids) CV risk factors as well as improvements in biochemical kidney disease outcomes (reduced urine protein excretion and improvements in serum creatinine and/or estimated glomerular filtration rate [eGFR]).
- **Study design**: all types of experimental (randomized and non-randomized), observational, cohort, and case-controlled designs.
- **Limits**: peer-reviewed journals and grey literature; publication date range (no limits); language (only publications in English language).

**Fig 1:**
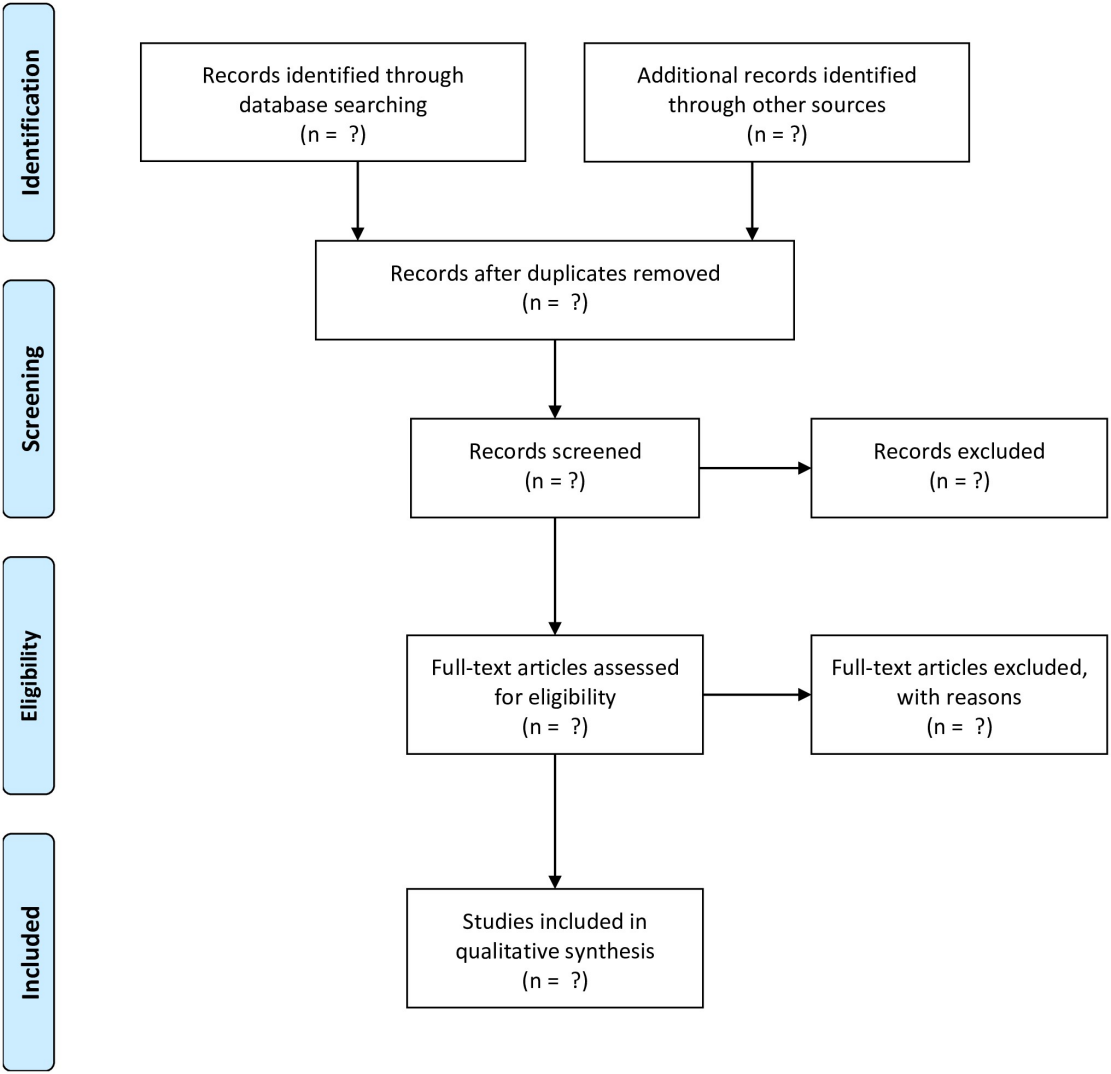
PRISMA-flow chart for study selection.

We will exclude studies if they were carried out in a mixed population that includes people of Indigenous groups, if pharmacological interventions were utilized (unless there is a clear separation between those who used pharmacological from non-pharmacological interventions), and studies conducted in paediatric populations (Fig 1).

### Definition of interventions

Any non-pharmacological interventions targeted to preventing or reducing the burden of CVD and/or CKD in Indigenous populations. This can be educational, telehealth, healthcare worker (e.g., nurse, pharmacist, community health worker, etc.), nutritional, exercise, organizational/facility-based, multifaceted, and culturally appropriate.

### Search strategy for databases

In developing this search strategy, we aimed at using a comprehensive review of the existing evidence base. We will identify studies through a detailed search (from inception) of the following bibliographic databases: Medline (Ovid), Embase (Ovid), Cochrane Library, CINAHL, ISI Web of Science and PsycINFO. We will also search grey literature using recommended resources in consultation with our medical Librarian. Thus, we will search ProQuest Dissertations & Theses Global, and Conference Proceedings Citation Index (Clarivate Analytics). We have developed the search strategy to be used in Medline (S1 Table) and will adapt this to be used in other databases. The search strategy will include subject headings, related terms and keywords necessary for the research question. We will use Boolean logic and operators (i.e., ‘AND’, ‘OR’, ‘NOT’) to combine and refine search terms. Additional hand searches will be carried out by tracking citations and through reference chaining of identified studies to identify studies from other Indigenous groups likely to have been missed by our search strategy.

### Data extraction

Results of the search will be collated in a Microsoft Excel database. We will follow recommended data charting methods [41] to systematically capture relevant details for included studies such as general study characteristics (author last name, study type, publication year, country of study, Indigenous group, income group, etc), study design (randomized controlled trials, observational, case-control study, cohort, etc) and type of intervention used (telehealth, healthcare worker, exercise, nutritional, culturally appropriate interventions, etc) (Table 2). We will also document outcomes from each study, including improved behavioural risk factors (smoking, alcohol consumption, dietary, and physical activity), improved clinical indices (BP and body weight), laboratory markers (any glycaemic index utilized [e.g., haemoglobin A1C [HbA1c] or blood sugar], lipids, kidney function [eGFR and/or urine protein]). All extracted data will be reviewed by 2 investigators for accuracy and completeness.

**Table 2:** Data extraction and charting for empirical literature sources.

Theme / subtheme	Description
**General details**	
• Author	Name of first author
• Year of publication	Year of study publication
• Country	Country where the study was performed
• Indigenous group	Name (identification) of the indigenous group (e.g. Canadian First Nations, Australian Aboriginal, etc)
• Sample size	What was the number of people involved in the study
**Study context**	
• Summary study aim	The aim of the study will be summarized
• Study design	Was the study an RCT, observational study, case-control or other
• Target Population	What was the study target population: general population, hypertensives, diabetics, etc
• Study intervention	Exercise, nutritional/diet-based, educational, telehealth, healthcare worker, organizational / facility-based, multi-faceted (if combination of interventions is used), and culturally appropriate interventions.
• Summary of study main findings	The main findings of the study will be summarized
• Summary of main outcomes	The main outcomes (CV and/or kidney-related) will be identified from included studies
• RE-AIM items	Components of the RE-AIM dimensions will be collected for analysis.

RE-AIM—Reach, Effectiveness, Adoption, Implementation, Maintenance; RCT—Randomized controlled trial; CV—cardiovascular.

### Collating, summarizing, and reporting of the results

Given our study aim is to assess the impact of interventions used to reduce CV and CKD risk in Indigenous communities, we will utilize the reach, effectiveness, adoption, implementation, and maintenance (RE-AIM) framework, developed by Glasgow et al [30] (http://www.re-aim.org/). This framework provides a practical means of evaluating health interventions (i.e., programs, policy, and practice) to assess the impacts of interventions and has primarily been used in studies focused on changing individual behaviours. Each dimension of the framework is briefly described below:

- **Reach** represents the number, proportion, and representativeness of people who are willing to participate in a given initiative, intervention, or program, and reasons why or why not **(Level: individual)**
- **Effectiveness** describes the impact of an intervention on important individual outcomes, including potential negative effects, including quality of life and economic outcomes; and variability across subgroups **(Level: individual)**
- **Adoption** is the proportion and representativeness of settings (e.g., health departments, or communities) that adopt a given policy or program and the reasons why **(Level: organizational / community)**
- **Implementation** refers to the extent to which a program or an intervention is consistently delivered as intended across different settings, staff and patients **(Level: organizational / community)**.
- **Maintenance** is the extent to which a program or policy becomes institutionalized or part of the routine organizational practices and policies. At the individual level, maintenance represents the long-term effects of a program on outcomes after 6 or more months after intervention contact **(Level: individual or organizational / community)**.

Using the RE-AIM framework (Table 3), we will summarize the reporting of each domain by intervention type from included studies. For each of the five RE-AIM dimensions, the presence or absence of indicators will be coded as “yes” or “no”. A total of 21 RE-AIM indicators will be coded, including indicators to describe reach (n = 5), efficacy/effectiveness (n = 4), adoption (n = 6), implementation (n = 3), and maintenance (n = 3). We will also assess RE-AIM reporting quality by the degree to which RE-AIM items were reported using the total frequency of the 21 RE-AIM items within each study. Thus, reporting of 0–7 items will be ranked as low, 8–14 as moderate, and 15–21 as high quality. There will be no quantitative synthesis of the data collected, however, we will provide a narrative description of primary studies and frequency counts and percentages across reported RE-AIM indicators.

**Table 3:** Measures to capture internal and external fidelity of community-based interventions to reduce cardiovascular and kidney disease from non-pharmacological interventions in Indigenous populations.

RE-AIM dimension	Definition	Metrics
***Reach*** into target communities	The absolute number of communities willing to participate in the program.	• Method to identify target population (database, community engagement)
		• Inclusion criteria
		• Exclusion criteria
		• Participation rate (number agreed to participate from those approached to participate)
		• Representativeness
*E****ffectiveness*** of the intervention	The impact of an intervention on key outcomes (process-based and clinical), including potential negative effects and economic outcomes.	• Results for at least one follow-up (follow up report)
		• Intent-to-treat analysis utilized (people treated with intervention / control)
		• Quality-of-life or potential negative outcomes (e.g. no significant decline)
		• Percent attrition (loss to follow up)
***Adoption*** by target communities	The number of communities, community leaders and PCPs who are willing to implement the program.	• Description of intervention location
		• Description of staff who delivered intervention
		• Method to identify staff who delivered intervention (target delivery agent)
		• Level of expertise of delivery agent
		• Inclusion/exclusion criteria of delivery agent or setting
		• Adoption rate of delivery agent or setting
***Implementation*** consistency, costs and adaptions made during program (intervention) delivery	Refers to fidelity to the program protocol and/or business case as well as the costs and adaptations made during the process of implementation.	• Intervention duration and frequency
		• Extent protocol (program) delivered as intended
		• Measures of cost of implementation
***Maintenance*** of intervention effects among individuals (patients and PCPs) and communities over time	The extent to which a program or policy becomes institutionalized or part of the routine organizational practices and policies.	• Assessed outcomes ≥6 months post intervention
		• Qualitative measure of individual-level maintenance
		• Measures of cost of maintenance

CKD—chronic kidney disease; PCP—primary care physicians; RE-AIM—Reach, Effectiveness, Adoption, Implementation, Maintenance.

### Patient and public involvement

Patients and public will not be involved at this stage of the project. However, we will consult Indigenous scholars to guide us with the development of the study including guidance on use of cultural interventions that are acceptable to Indigenous groups for reduction of CV and kidney diseases. We will synthesize publicly available publications which reported on non-pharmacological interventions to reduce CV and kidney disease in Indigenous populations.

### Ethics and dissemination

Ethical approval will not be needed for this study as data used will be extracted from already published studies. Our dissemination strategy will use traditional approaches, including open-access peer-reviewed publication(s), scientific presentations, and a report. Also, we will disseminate our findings to the government—Alberta Health, Alberta Health Service (AHS), Indigenous leaders, and Federal government and the First Nations and Inuit Health Branch (FNIHB) Canada.

## Strengths and limitations of this study

This study will assess the impact of non-pharmacological interventions for reducing cardiovascular (CV) and kidney-related outcomes in remote dwelling Indigenous populations using available publications from various databases from inception. Although the role of pharmacological interventions has often been studied regarding reduction of CV and kidney disease, this study will focus on use on non-pharmacological interventions in remote Indigenous populations who are often underserved in terms of availability of healthcare services. Also, using the RE-AIM framework to evaluate the effects of interventions will be useful for highlighting internal and external fidelity of reporting across studies in Indigenous populations. A potential limitation of this study could be low reporting of outcomes from identified studies, especially outcomes that are kidney disease related following use of non-pharmacological interventions. This may likely limit the interpretation and applicability of our data. Another limitation of the study could relate to the search criteria being focussed predominantly on Indigenous populations in North America and Western Pacific countries. However, it is well known that most published studies on Indigenous populations have largely been conducted in these groups, given their high-risk for cardio-metabolic conditions. We will mitigate this limitation by conducting further hand searches for studies that may enable us to identify studies in other Indigenous groups.

## Supporting information

S1 Table MEDLINE search strategy. (PDF)

S1 File (PDF)
