# Supplementary material for: The impact of community-based non-pharmacological interventions on cardiovascular and kidney disease outcomes in remote dwelling Indigenous communities: A scoping review protocol
Source: PLoS One. 2022 Jun 10;17(6):e0269839. doi: 10.1371/journal.pone.0269839 (PMC9187124; doi:10.1371/journal.pone.0269839)
Supplement: S1 Table — (PDF) [file pone.0269839.s001.pdf]

Supplementary Table S1: MEDLINE search strategy

1 exp American Native Continental Ancestry Group/ (21703)

2 Indigenous Peoples/ (379)

3 United States Indian Health Service/ (583)

4 exp Health Services, Indigenous/ (3415)

5 (Athapaskan or Saulteaux or Wakashan or Cree or Dene or Inuit or Inuk or Inuvialuit* or Haida or Ktunaxa or Tsimshian or Gitsxan or Nisga'a or Haisla or Heiltsuk or Oweenkeno or Kwakwaka'wakw or Nuu chah nulth or Tsilhqot'in or Dakelh or Wet'suwet'en or Sekani or Dunne-za or Dene or Tahltan or Kaska or Tagish or Tutchone or Nuxalk or Salish or Stl'atlimc or Nlaka'pamux or Okanagan or Sec wepmc or Tlingit or Anishinaabe or Blackfoot or Nakoda or Tasttine or Tsuu T'inia or Gwich'in or Tagish or Tutchone or Algonquin or Nipissing or Ojibwa or Potawatomi or Innu or Maliseet or Mi'kmaq or Micmac or Passamaquoddy or Haudenosaunee or Cayuga or Oneida or Onodaga or Seneca or Tuscarora or Wyandot).tw,kf. [CANADA-SPECIFIC TRIBES] (3970)

6 (Abenaki* or Arapaho* or Assiniboine or Beothuk or Blackfeet of Blackfoot or Cabazon* or Cheyenne* or Cadoo* or Cherokee* or Chicksaw or Chickasaw or Chippewa or Chitimacha* or Choctaw* or Cocopah* or Coeur-D'alene or Comanche* or Coushatta or Duwamish or Elwha* or Flathead* or Goshute* or Ho-Chunk* or Hopi or Hopis or Hoopa* or Jatibonicu or Jumano* or Kalapuya* or Kanaka-Maoli or Kiowa* or Kootenai* or Lakota or Lemhi* or Makah* or Metis or Menominee* or Miccosukee* or Mi'kmaq or Mohegan* or Mechoopda* or Mohawk* or Muscogee* or Nakota or Navajo* or Nez-Perce or Nez-Pierce or Oneida* or O'Odham or Osage* or Passamaquoddy or Pawnee* or Pend-D'oreille or Pacific-Island* or Pequot* or Pima or Pimas or Pomo or Pomos or Potawatomi* or Pueblo* or Quinault* or Salish or Saponi or Saponis or Seminole* or Shawnee* or Shoshone* or Siletz or Sioux or S'klallam or Suquamish or Taino or Tainos or Tlingit* or Tohono or Tunica-Biloxi or Tunicas or Tuscarora or Umatilla* or Umpqua* or Ute or Utes or Waccamaw* or Wampanoag* or Washoe* or Wiyot* or Yakama*).tw,kf. [USA-BASED NATIVE TRIBES] (12477)

7 (Anangu or Anindilyakwa or Arrernte or Bininj or Goorie or Gunggari or Iwi or Koori or Koorie or Luritja or M#ori or Murri or Muruwari or Ngunnawal or Ng#puhi or Noongar or Nunga or Palawah or Pallawah or Pitjantjatjara or Tangata-Whenua* or Tiwi or Torres-Strait-Island* or Wangai or Warlpiri or Yamatji or Yolngu).tw,kf. [AUSTRALIAN/NEW ZEALAND TRIBES] (5733)

8 (Aymara or Caxixo or Guaran# or Kambeba or Lencas or Mapuche or Mayan* or Mestizo* or Minea-Gerais or Miskito* or Pataxo or Quechua).tw,kf. [SOUTH AMERICAN INDIGENOUS TRIBES] (3029)

9 (Bushman* or Himba or Ndebele or Maasai or Samburu or Zulu*).tw,kf. [AFRICAN INDIGENOUS TRIBES] (1162)

10 (Sami or Samis or Saami or Lapland* or Lapp?).tw,kf. (1540)

11 aboriginal*.tw,kf. (9306)

12 circumpolar*.tw,kf. (1019)

13 indigenous*.tw,kf. (34724)

14 metis.tw,kf. (377)

15 red-road.tw,kf. (5)

16 on-reserve.tw,kf. (204)

17 off-reserve.tw,kf. (105)

18 (First-nation or First-Nations* or First-people?).tw,kf. (2211)

19 (American Indian* or Amerindian*).tw,kf. (9001)

20 (rural adj3 (Indian* or Native*)).tw,kf. (1142)

21 (Native* adj1 (man or men or woman or women or boy* or girl* or adolescent* or youth? or person* or adult or people* or Indian* or Nation or band? or Hawaiian*)).tw,kf. (3213)

22 ((Indian* or Native*) adj2 (tribe* or tribal or reserve* or reservation*)).tw,kf. (1297)

23 or/1-22 [MeSH & KEYWORDS FOR INDIGENOUS POPULATIONS; MODIFIED FILTER FROM UNIVERSITY OF ALBERTA] (86289)

24 community health services/mt, og (10252)

25 ((community care or community health*) adj5 (outreach or program* or service*)).tw,kf. (5247)

26 preventive health services/mt, og (4604)

27 (preventive adj5 (program* or service*)).tw,kf. (14378)

28 primary prevention/mt, og (8384)

29 ((diseas* adj2 prevent*) or (primary adj2 prevent*)).tw,kf. (68301)

30 delivery of health care/mt, og (26804)

31 ((care or healthcare or health care) adj3 (deliver* or service* or system*)).tw,kf. (202276)

32 health promotion/mt, og (33079)

33 ((health* or healthcare* or wellness) adj3 (campaign* or program* or promot*)).tw,kf. (124396)

34 ((outreach adj2 campaign*) or (outreach adj program*)).tw,kf. (2048)

35 early medical intervention/mt, og (1061)

36 ((early adj2 diagnos*) or (early adj2 identif*) or (early adj2 intervention*)).tw,kf. (161621)

37 community-institutional relations/ (10742)

38 ((community* adj3 outreach) or (community* adj3 relation*)).tw,kf. (5376)

39 (community-based or community-led or health* worker-led or nurse-led or practitioner*-led).tw,kf. (68609)

40 mass screening/ (106066)

41 ((mass or mobile) adj3 (screen* or test*)).tw,kf. (13568)

42 (point-of-care adj3 (screen* or test*)).tw,kf. (7615)

43 telemedicine/ (26357)

44 (tele-health* or telehealth* or tele-medicin* or telemedicin*).tw,kf. (20704)

45 ((instant messaging adj2 remind*) or (short message adj3 remind*) or (sms adj2 remind*) or (text* adj2 remind*)).tw,kf. (778)

46 counseling/ (36517)

47 counsel*.tw,kf. (115407)

48 house calls/ (3724)

49 ((house adj2 call*) or (home* adj2 visit*)).tw,kf. (11524)

50 health services, indigenous/ (3415)

51 (improve* or improving).ti,kf. (323241)

52 ("impact of" or "impact on").ti,kf. (242968)

53 intervention?.ti. (146614)

54 prevent*.ti,kf. (347107)

55 (program or programs or programme or programmes).ti,kf. (206650)

56 (reduce? or reducing or reduct*).ti,kf. (356386)

57 (strategies or strategy).ti,kf. (156130)

58 or/24-57 (2386587)

59 exp cardiovascular disease/di, dg, dh, dt, pc, th (1274679)

60 (cardiovasc* adj3 (diseas* or health or outcome? or risk?)).tw,kf. (273075)

61 CVD.tw,kf. (38879)

62 exp hypertension/ (258341)

63 hypertens*.tw,kf. (455044)

64 ((control* or elevat* or chang* or high or lower or reduce? or reduct*) adj2 blood pressur*).tw,kf. (76451)

65 ((control* or elevat* or chang* or high or lower or reduce? or reduct*) adj2 bp).tw,kf. (16711)

66 exp kidney diseases/di, dg, dh, dt, pc, th (233411)

67 exp renal insufficiency, chronic/ (118161)

68 (kidney disease? or kidney failure or kidney insuffic*).tw,kf. (98531)

69 (renal disease? or renal failure or renal insuffic*).tw,kf. (168882)

70 (CKF or CKD or CRD or CRF or ESKD or ESKF or ESRD or ESRF).tw,kf. (67438)

71 diabetic nephropathies/ (25933)

72 (diabetic glomerulosclerosis or diabetic nephrop* or intracapillary glomerulosclerosis or nodular glomerulosclerosis or kimmelstiel Wilson).tw,kf. (20625)

73 diabetes mellitus, type 2/ (137708)

74 diabet*.tw,kf. (666271)

75 T2DM.tw,kf. (23081)

76 exp smoking/ (150252)

77 (cigar? or cigarette* or e-cig* or marijuana or smoker* or smoking or tobacco or vape or vapes or vaping).tw,kf. (347658)

78 exp alcohol drinking/ (70639)

79 exp alcohol-induced disorders/ (26266)

80 (alcoholic* or alcoholism*).tw,kf. (90678)

81 (alcohol* adj3 (abus* or addict* or consum* or depend* or disorder* or drink* or habit* or intoxicat* or problem*)).tw,kf. (116820)

82 overweight/ (25984)

83 exp obesity/ (219829)

84 (obese or obesity or over-weight or overweight).tw,kf. (339104)

85 ((los* adj1 weight) or weight loss).tw,kf. (96537)

86 hypercholesterolemia/ (26042)

87 hypercholesterol*.tw,kf. (35950)

88 ((elevated or high or lower or reduc*) adj3 (cholesterol* or LDL or HDL)).tw,kf. (86523)

89 sedentary behavior/ (10304)

90 (sedentary behav* or sedentary life* or physical inactiv*).tw,kf. (20095)

91 or/59-90 (3420248)

92 23 and 58 and 91 (3859)

93 animals/ not (humans/ and animals/) (4754039)

94 92 not 93 (3837)
